# Power relations and knowledge of neonatal teams in the Kangaroo Mother Care implementation and dissemination^*^

**DOI:** 10.1590/1980-220X-REEUSP-2022-0200en

**Published:** 2022-11-04

**Authors:** Luana Claudia dos Passos Aires, Maria Itayra Padilha, Evangelia Kotzias Atherino dos Santos, Zeni Carvalho Lamy, Maria Lígia dos Reis Bellaguarda, Isadora Ferrante Boscoli de Oliveira Alves, Rosiane da Rosa, Roberta Costa

**Affiliations:** 1 Instituto Federal de Santa Catarina, Joinville, SC, Brazil.; 2 Universidade Federal de Santa Catarina, Florianópolis, SC, Brazil.; 3 Universidade Federal do Maranhão, São Luís, MA, Brazil.

**Keywords:** Patient Care Team, Kangaroo-Mother Care Method, Infant, Newborn, Health Policy, Power, Psychological, Neonatal Nursing, Grupo de Atención al Paciente, Método Madre-Canguro, Recién Nacido, Política de Salud, Poder Psicológico, Enfermería Neonatal, Equipe de Assistência ao Paciente, Método Canguru, Recém-Nascido, Política de Saúde, Poder Psicológico, Enfermagem Neonatal

## Abstract

**Objective:**

to analyze the power relations and knowledge among health teams that permeate the Kangaroo Mother Care implementation and dissemination in the state of Santa Catarina.

**Method:**

socio-historical qualitative research, carried out in the state of Santa Catarina, from January to November 2019, based on interviews with 12 health professionals. Data were analyzed in the light of Foucault’s genealogical proposal, with the help of Atlas.ti Cloud^®^.

**Results:**

the relationships of neonatal team members strengthened Kangaroo Mother Care actions in the state, articulating services and favoring health professionals’ autonomy. However, Kangaroo nurses stand out in this process, and the hegemonic medical discourse often still represses the other professional categories.

**Conclusion:**

professionals develop strategies to negotiate changes in the practice of care, moving between the plots of power and knowledge, sometimes exercising it, sometimes being passive to it.

## RESUMO

**Objetivo:**

analisar as relações de poder e saber, entre a equipe de saúde, que permeiam a implantação e disseminação do Método Canguru no estado de Santa Catarina.

**Método:**

pesquisa sócio-histórica, com abordagem qualitativa, realizada no estado de Santa Catarina, no período de janeiro a novembro de 2019, a partir de entrevistas com 12 profissionais de saúde. Os dados foram analisados à luz da proposta genealógica de Foucault, com auxílio do *software Atlas.ti Cloud* ^®^.

**Resultados:**

as relações da equipe neonatal fortaleceram as ações do Método Canguru no estado, articulando os serviços e favorecendo a autonomia dos profissionais de saúde. Entretanto, a enfermeira Canguru se destaca nesse processo, e o discurso médico hegemônico, muitas vezes, ainda reprime as demais categorias profissionais.

**Conclusão:**

os profissionais desenvolvem estratégias para negociar as mudanças na prática do cuidado, transitando entre as tramas do poder e saber, ora o exercendo, ora sendo passivo a ele.

## RESUMEN

**Objetivo:**

analizar las relaciones de poder y saber, entre el equipo de salud, que permean la implementación y difusión del Método Madre-Canguro en el estado de Santa Catarina.

**Método:**

investigación sociohistórica, con enfoque cualitativo, realizada en el estado de Santa Catarina, de enero a noviembre de 2019, a partir de entrevistas con 12 profesionales de la salud. Los datos fueron analizados a la luz de la propuesta genealógica de Foucault, con la ayuda del software Atlas.ti Cloud^®^.

**Resultados:**

las relaciones del equipo neonatal fortalecieron las acciones del Método Madre-Canguro en el estado, articulando los servicios y favoreciendo la autonomía de los profesionales de la salud. Sin embargo, la enfermera canguro se destaca en este proceso, y el discurso médico hegemónico muchas veces aún reprime a las demás categorías profesionales.

**Conclusión:**

los profesionales desarrollan estrategias para negociar cambios en la práctica del cuidado, moviéndose entre las tramas del poder y del saber, a veces ejerciendo, a veces siendo pasivos frente a él.

## INTRODUCTION

The Kangaroo Mother Care (KMC) implementation as a public health policy in Brazil took place with the launch of the Norm for Humanized Care for Low Weight Newborns–KMC (NAHRNBP–KMC – *Norma de Atenção Humanizada ao Recém-Nascido de Baixo Peso*), through Ordinance 693/MO^(1)^. KMC is a model of care that contradicts the hitherto technocratic and curative model. As it is mainly related to hospitalization cost reduction, it suffered a series of criticisms since its implementation, mainly by developed countries, who considered it an alternative for third world countries^(2)^. The biomedical care conception transformation was procedural and slow. In Brazil, the implementation of this model seeks to associate the use of this soft technology with the hard technologies in neonatal intensive care^(3–5)^.

One of the pillars of KMC in Brazil is the practice of early skin-to-skin contact between mother and newborn. In many countries, KMC is just this practice (kangaroo position). Evidence indicates that skin-to-skin contact is a safe, soft technology that should be prioritized in low birth weight preterm infant care, compared to conventional neonatal care, as it significantly reduces the risk of mortality, increasing weight gain and rates of exclusive breastfeeding at hospital discharge^(3,6)^.

In Brazil, as a public health policy, there is a more comprehensive understanding of the method. Thus, it involves a set of actions made available in various materials, regulations and national ordinances, which integrate care for newborns and their families^(1,2)^.

The studies that approach KMC in Brazil focus on the family, on health professionals and on the three stages of the method, pointing out benefits in breastfeeding, family bond, infants prognosis. On the other hand, they bring up the difficulties of its implementation, despite all the training carried out, and point to a significant deficit of neonatal beds throughout the country^(3,7,8)^.

However, it is important to highlight that there is a gap in relation to published studies that bring the historical perspective of KMC implementation in Brazil. KMC’s trajectory in the country began in 1991, at the *Hospital Estadual Guilherme Álvaro*, in Santos (SP), and then at *Instituto Materno Infantil de Pernambuco* (IMIP), in Recife, in 1994^(1,2)^.

In order to intensify the implementation of the method (year 2000) throughout the national territory, considering the different cultural, social and economic scenarios of the country, National Reference Centers (NRC) were created. The state of Santa Catarina (SC) was representative in this scenario, when the *Hospital Universitário Professor Polydoro Ernani de São Thiago*, *Universidade Federal de Santa Catarina* (HU/UFSC), in Florianópolis, was chosen as one of the NRC^(2)^. Subsequently, based on the movement to strengthen and disseminate the KMC (year 2008), with a view to decentralization, the MoH requested that each State Health Department nominate a service to be a State Reference Center (SRC) for KMC. Thus, 27 maternity hospitals were selected, one in each Federative Unit and the Federal District, with the state of SC represented by *Maternidade Darcy Vargas* (MDV), in Joinville^(2)^. The choice of these services was due to their humanization trajectories in neonatal care, as indicated by the State Health Departments.

Despite all the MoH’s and reference center professionals’ effort and work for disseminating and strengthening the KMC, there are still few Neonatal Units (NU) that managed to implement the three stages, according to the proposed method. Available public data indicate only eight services in the state of SC with a Kangaroo Care Unit (UCINCa) registered^(9)^. The literature points to a scenario of resistance, observed, in practice, for implementing the method in the country, as barriers generated by the team itself, work processes, management, lack of resources, among others^(3,4,5)^.

This investigation aimed to analyze the power relations and knowledge among health teams that permeate the KMC implementation and dissemination in the state of SC.

## METHOD

### Study Design

This is qualitative socio-historical research, based on Michel Foucault’s theoretical-philosophical aspects^(10)^, based on thematic oral history (OH)^(11,12)^. From a historical analysis, it is possible to give voice to the professionals who worked in the method implementation and dissemination in SC. In this historical process of construction of the Brazilian proposal for KMC, its implementation begins in 1996, when there are records of the first actions to humanize neonatal care in the state, being consolidated in 2000 with the launch of the Guidance Standard for the Implementation of Kangaroo Mother Care, aimed at promoting humanized care for low birth weight newborns^(1)^. The strengthening and dissemination were formalized in 2008, based on the MoH proposal for the decentralization of actions, and this process continues to this day.

To describe the methodological design of this study, seeking to meet international quality criteria for qualitative research, we used the COREQ checklist (COnsolidated criteria for REporting Qualitative research)^(13)^.

### Participants

Study participants are oral sources, consisting of 12 professionals from neonatal teams and/or health managers. We included consultants/tutors/health managers of a health team (nurse, social worker, physiotherapist, occupational therapist, physician, psychologist) who have participated in the transformation of the care scenario based on the KMC proposal, considering the period from 1996 to 2019 We exclude professionals who have worked for less than three years in the method.

### Local

This study was carried out in SC, southern state of Brazil, which has eight health institutions with UCINCa beds, qualified (27 beds) in different municipalities. Considering the historical approach, the time frame is delimited by the period in which actions related to KMC at the HU/UFSC began (1996), ending this historical marker in 2019, when the first state meeting of SC tutors took place, after almost 20 years of implementation and dissemination process of the method in the state.

### Data Collection and Organization

Data collection took place from January to November 2019, and the starting point for contacting the study participants was the HU/UFSC CRN, using the snowball technique to identify the other study participants^(14)^. We applied an interview guided by a guide-script (an instrument that was previously assessed by three expert researchers in the area), containing questions about how the process of KMC implementation and dissemination in the state occurred.

The invitation to participate in the study was carried out in three ways: in person, by telephone contact from the institution or e-mail, and the interviews were scheduled according to each participant’s availability. The interviews were conducted by a single researcher, all of which were audio-recorded. Transcription was carried out in full, respecting the criteria regarding transcription and transcreation fidelity. The mean duration of each interview was 60 minutes. After transcribed, were sent to the participants via e-mail, in order to be validated by the interviewees and formally authorized for use in the study after signing the Oral Testimony Rights Assignment Letter. Data collection ceased from data saturation, when the interviews did not add new data to the study^(15–16)^. A field diary was also used, in which the researcher’s perceptions about the interviews and insights during the investigative process were recorded.

### Data Analysis and Treatment

Data analysis was carried out in the light of genealogical analysis, proposed by Foucault. After transcribing and validating the interviews, the material was organized using the free online version of Atlas.ti Cloud^®^. In this instrument, the interviews were inserted separately, with text skimming. Then, in the material exploration stage, participants’ speeches were highlighted in the interviews themselves in thematic groupings, or, as called in this software, codes, which resulted in 26 coding items. After completing the code selection, each code was read and reread separately, allowing an overview of the different versions of the story, told by the characters who helped to build it, allowing to choose the significant speeches. The data corpus enabled the provisional discursive formations, which were organized in a Microsoft Word^®^ document, which gave rise to analytical units according to objectives and theoretical framework.

### Ethical Aspects

The investigation followed Resolution 466/12, and was approved by the Research Ethics Committee, in 2018, under number 3,091,461. Considering the context of the historical research, participants are identified by their first name and profession, this aspect being formally authorized by signing the Informed Consent Form (ICF) and Oral Testimony Rights Assignment Letter.

## RESULTS

Twelve professionals from a multidisciplinary health team (five nurses, four physicians, a psychologist, an occupational therapist and a social worker) participated in the study, who participated/participate in the process of KMC implementation and dissemination in the state of SC. These professionals represented six SC health institutions in four municipalities. From data analysis and interpretation from Michel Foucault’s perspective, the following analytical units were listed: *Relationships strengthen Kangaroo Mother Care; Kangaroo nurses;* and *The hegemonic medical discourse*.

### Relationships Strengthen Kangaroo Mother Care

Professional relationships were pointed out as something positive, which favored implementing the method from the expansion of inter-institutional contacts. An important milestone brought by many study participants to strengthen the KMC in the country and, consequently, in the state of SC, was the holding of the First National Conference on the “Kangaroo Mother” (as the method was previously called).

Technical visits were carried out by KMC consultants and tutors as a MoH strategy to strengthen the method implementation and dissemination, strengthening ties between professionals and proposing solutions in practice.

*So, this was the event that triggered in March 1999 several contacts with the BNDES, with Dr. Geisy, with Hector Martinhez. And Dr. Geisy, who is a neonatologist, I was delighted and invited her to come in April to give a lecture here at the UH to present the experience of IMIP*. (...) *In the meantime, until December 1999*, *I was already making connections with the management here, with the rectory* (...) *who supported the BNDES to carry out the physical renovation* (Zaira/Psychologist).

*So, as a goal for this year, it would be to strengthen these ties between the units, because, sometimes, like, “ah, I can’t start the Kangaroo Unit, because I don’t have adequate space”. Our space is not the best either, but we start, “my management doesn’t believe that giving up an accommodation room to deliver something that maybe not even right...”, look at us too, but say that in others hospitals work, understand?* (...) *we take a little bit of what we do here and we take a little bit of what they do there, and then we mix it up and I think there is a new model like this* (Fernanda/Nurse).

For KMC actions to work in the institutions, the multidisciplinary team’s work in the daily routine of care was essential.

*All professionals need to be involved and active, encouraging touch, care with ambience, among other things*. (...) *without the multidisciplinary team, without the commitment of the nursing, the psychologist, the physical therapist, we cannot motivate the rest of the team to maintain the method. If someone says something negative during a hospital stay, you could lose all your working time there* (Osmar/Physician).

*Humanization actions depend on the entire team “everyone is responsible for humanization”, including to monitor and supervise their co-workers*. (...) *it is not just the nurse who is responsible. Everyone is responsible for humanization”* (Zaira/Psychologist).

### Kangaroo Nurses

The role of nurses was strongly highlighted by participants as significant and differential for the feasibility of the method’s actions. This active nurse profile grouped certain characteristics and made many participants recognize them as “Kangaroo nurses”.

Nurses had to go beyond professional competences as coordinators of nursing teams and responsible for systematization of nursing care, starting to disseminate, in practice, the KMC principles, encouraging other professionals to change their practices. The multidisciplinary team highlights the importance of a Kangaroo nurse for the effectiveness of the method in the institution.

(...) *Cintia* (Nurse) *was an extremely detailed person in the smallest things, which, for me, everything was fine, cute, normal, she showed us that in those small details was all the difference* (...) (Zaira/Social Worker).

(...) *I had a difference. It’s true* (...) *and, in this case, I was the nurse that involved me the most. I had a more humanized practice; I was more attuned to this humanization thing* (...) *I used to take care. And I took care of the team too.* (...) *the bond that we made for the mother and the infants was also strong for us, you know* (smiles) (Sônia/Nurse).

(...) *in terms of care, that’s where I really started, it’s been two or three years since I took charge of the Kangaroo. I saw that the nurse needed to take the lead, that if I didn’t take the lead, it wouldn’t happen, I wouldn’t leave. And that’s where I embraced the cause, and Dr. Osmar was a person who helped me a lot in this regard*. (...) *so I tell you, Kangaroo Mother Care is from the nurse. If the nurse does not embrace the cause, it cannot be effective. Does not work. It’s no use waiting for others to do it, because they won’t do it. You have to go after it, and it’s an ant job* (laughs). (...) *everywhere, I think it is so. If they don’t put nurses together, it doesn’t work* (...) *what motivated me to embrace this cause, was seeing that it depended a lot on us* (...) (Camila/Nurse).

(...) *we then carried out a pre- and post-intervention work, assessing humanization practices in the Neonatal Unit. Infants weighing 2,000 g were assessed, hospitalized in two different periods, immediately before and after hiring the nurse. We saw that the use of Kangaroos increased from 49% to 70%, and that was significant. Showing that the nurse is fundamental, someone who is there on a day-to-day basis and encouraging the employees, and demanding from the employees, and justifying and training*. (...) *what we always worked on was showing that the nurse’s role is fundamental in the NEO* (Maria Beatriz/Physician).

### The Hegemonic Medical Discourse

Some testimonies point to medical hegemony in relationships, such as the release to the kangaroo position, which is often still conditioned on the authorization/prescription of pediatricians/neonatologists, and not on the scientific evidence that justifies such a practice. This medical “power”, however, transits between the other health professionals and the parents, who, when understanding KMC as something beneficial to infants, do it even without authorization, in secrecy and “behind the curtains”. Expanding other professionals’ autonomy to place newborns in a kangaroo position can provide opportunities for this action to be performed more often.

It is interesting to reflect that, although initially conditioned to professional physicians to perform the kangaroo position, with regard to involvement in tutoring actions for disseminating the method throughout the state, these professionals were little involved.

*But we have a lot of resistance from a physician here in our ICU*. (...) *a resistance that stops us really often* (...) *the nurses are a little scared, because, as he is the coordinator, they are afraid*. (...) *because we don’t have a public examination here. I think that, if we had, we would have much more autonomy to do this kangaroo issue more effectively with regard to this specific physician* (...) *you have to ask to see if you can put* (infants in kangaroo position). *Even if, sometimes, newborns are in ambient air, we have to keep asking, and it’s annoying, because it takes some of your autonomy from the nurse’s care* (...) *nurses know how far he can go. (...) the intubated, we have a fight with him. There’s a NEO physician of ours* (...) *she says to us, “I’ll put it on, but if you say...”. Because she ends up taking it too, he is also her coordinator. She says, “Let’s put it on, but, mom, you can’t say it”... we close the curtain, put it on and pray* (...) *that everything goes well. Don’t get sick, nothing, and girls don’t count* (...) (Camila/Nurse).

*I think the people who can be said to have worn the clothes of warriors are few, very few. They were always the same ones arguing, trying to do things, searching, and the others paddling along*. (...) *physicians, the vast majority also fled the representativeness of getting involved with something else* (Márcia/Nurse).

## DISCUSSION

We identified that the various forms of professional relations that were established during training, qualification and technical visits strengthened the KMC implementation and dissemination actions in the state. This allowed the articulation of services with important characters, such as MS consultants. A study carried out in Rio de Janeiro also points to positive influence of interaction between professionals for compliance with good practices of humanization in NU^(17)^.

The importance of multidisciplinary teams in the development and monitoring of the method’s actions in health services stands out. This shared responsibility in care favors all professional categories’ autonomy. In this situation, all professionals from the multidisciplinary team working in KMC have scientific knowledge and, consequently, the power to put it into practice in the state. Also, in this relationship of exchange of experiences, the role of consultants and state tutors of the method stands out, important characters in this historical clipping of implementation and dissemination of the method in SC. They are professionals who have emerged from the knowledge that, in turn, they hold power in the dissemination of good practices, being respected for the representativeness they exercise. According to Michel Foucault’s theoretical framework used in this study, every human grouping will always be surrounded by power relations^(11,18,19,20)^, in which knowledge is not neutral, but a political act^(21)^. Health professionals, individuals who take care of others, in general, are inserted in a network of powers that, in the practice of care, give them this authority, due to their technical-scientific knowledge^(20,22,23)^.

Power is seen as a concept that seeks to understand how social practices occur^(10)^. This positive character of power corresponds also to something that is exercised more than what is possessed^(10)^. Power is vertical, so it dissipates horizontally in micropower, i.e., it dissipates among the entire multidisciplinary team. Lack of teamwork makes it difficult to implement the^(24,25)^ method. Each team professional has a role of multiplier, potentially influencing (dis)continuity of KMC^(17)^.

A team that is safe and aware of KMC more easily overcomes the barriers related to the weaknesses experienced in most hospitals to work in KMC^(3,6)^. Strategies used by nurses, such as training and awareness-raising, can guarantee the strengthening of KMC, and the continuing education of health teams has decreased the barriers and improved the care process in the NU^(25)^.

It is also worth mentioning the power exercised by the “Kangaroo nurses” (so called by the participants of this study), those professionals who are beyond the average, who see beyond the needs of infants and their families and who are concerned with the small details, guaranteeing comprehensive care for these subjects. The fundamental importance of Kangaroo nurses is observed in participants’ speeches, and without this figure at the forefront of the method’s actions, risks are identified for its effective implementation in the services. For the emancipation of nurses in their work in the health area, knowledge, study and empowerment are necessary, allied to a social and political transformation that has been taking place in the profession and in the nursing care model, from curative and biomedical actions to health promotion^(26)^. In this sense, nurses stood out playing a role of organizer of the work process and disseminating in the team the need to implement good practices, thus exercising their power in the team.

The literature recognizes that, among health professionals, nurses stands out, with their KMC multiplier profile, while they are faced with the care and management challenges in the NU^(3,27)^. Nurses are increasingly active in the design of public health policies, requiring the empowerment of the class and more critical pedagogical curricula that strengthen the category^(26)^. “Kangaroo nurses” exert a subtle power over the other multidisciplinary team members, a power that, in a way, we can classify as disciplinary power. In this context, nurses develop sovereign discipline strategies, based on obedience, standardization and regulation of the actions of other professionals in KMC^(18)^.

We understand, however, that for the consolidation of KMC in health services of the state, we need to have “kangaroo teams”, of which all the characters involved understand and feel invited to put into practice the method’s actions, this action not being the exclusive or predominant responsibility of a certain category. Thus, power is exercised by different people in different contexts of daily care, causing power to circulate among professionals.

Finally, in “The hegemonic medical discourse”, we identified that, at times, these professionals have the power, as in the cases of releasing or not the infants to the kangaroo position. However, this power circulates when other professionals, even without medical “authorization”, put infants in skin-to-skin contact with their mother. It is questionable, however, why multidisciplinary team professionals, although endowed with knowledge about the benefits of the method for infants and their families, still often perform it informally, hidden, without appropriating the power entrusted to them by knowledge to initiate confrontation, struggle and resistance strategies.

These difficulties are observed throughout the state of SC, both in public and private services; however, professionals in private services justify their omission and/or passivity for fear of losing their jobs. This medical hegemony is historically justified in hospital organizations and health services, considering that preterm infant care is predominantly focused on the biomedical and curative model^(3,23)^. The medicalization of society implied hierarchical and disciplinary relationships, in which the supremacy of scientific physician knowledge (biopower) in the therapeutic decision is observed, basing practices and protocols^(21,23,27,28)^. In this organizational model, power relations transit between those who prescribe care and those who execute it^(21)^.

In this centrality of physician knowledge, blaming and frightening behaviors are verified in professionals^(27)^. This controlling action of medical surveillance over the work of colleagues hinders other health professionals’ autonomy^(21)^. In a recent study, nurses reported that they have their power of decision and action limited by medical restrictions, facing resistance to the practice of KMC^(6)^. It is extremely important to expand the multidisciplinary team’s autonomy so that this care is formalized in care practice and performed more often. Training and awareness-raising courses to the method were and have been fundamental for coping with these resistances.

The term “power” from Latin *possum*, means to be able to have the right to deliberate, act and command, exercise authority, impose obedience, sovereignty, or empire of a given circumstance. In the daily routine of hospital work, there are micropower that circulate health professionals^(21)^. This study corroborates Foucault’s framework, when analyzing these micropower, which act in the professional relationships of tutors/consultants and method managers. A power that emancipates empowers, but that is not static, it is dynamic and circulates among health professionals.

The use of Foucault’s ideas and conceptions refers to compliance with a robust theoretical-philosophical framework, which constantly invites to the reflection of everyday life and routine, helping to identify the plots of power/knowledge^(10)^. Foucault’s genealogical analysis of this study is an attitude towards historical facts, a perspective that allows us to seek the construction of a historical knowledge of struggles from the search for beginnings, however discontinuous, from the study of the microphysics of relationships: biopower and biopolitics^(11,29,30)^.

A limitation of this study is the selection of key characters using the snowball technique, in which engaged professionals who were influential in the process of implementing and disseminating the state were mainly indicated. Perhaps, by expanding the approach to more top professionals, it will be possible to identify why so many weaknesses are still found in the state for implementing the KMC.

Much has been researched about KMC in Brazil and in the world, however without the concern with the historical perspective. Considering the more than 20 years of this public policy instituted in the country, this type of approach in the light of Foucault’s framework highlights the roots of the difficulties faced over the years, to ensure its implementation and dissemination. From the understanding of how these power relations-knowledge occur between professionals, it is possible to outline strategies, in which this study highlights the importance of nurses for implementing this policy.

## CONCLUSION

By analyzing the power relations and knowledge among health teams, which permeate the KMC implementation and dissemination in SC, we identified that exchange of experiences strengthened the KMC actions in the state from service articulation. The performance of multidisciplinary teams is fundamental in the development and monitoring of the method’s actions. This joint work confers shared responsibility for care among health professionals, favoring their autonomy.

Among the various professional categories, “Kangaroo nurses” stands out in the NU of SC as those professionals who are concerned with small details and guarantee comprehensive care for infants and their families, developing strategies of subtle power between the team/family, which is a fundamental part for the effectiveness of KMC in health services.

The historical context of hospital organizations and health services still brings reports that mark history with the realization of the “hegemonic medical discourse”, which still represses the other professional categories. This study points out that, in the daily life of NU, different power relations are established and that each professional develops their strategies to negotiate changes in the practice of care, moving between the plots of power and knowledge, sometimes exercising it, sometimes being passive to it.
